# Hospital Management

**Published:** 1911-09

**Authors:** 


**Hospital Management.** A Handbook for Hospital Trustees, Superintendents, Training School Principals and Physicians. By **Charlotte A. Aikens,** Author of "Hospital Training-School Methods and the Head Nurse." 12mo of 488 pages, illustrated. Philadelphia and London: W. B. Saunders Company. 1911. (Cloth, $3.00 net.)

This book of about 500 pages is one of the most valuable contributions to the literature on hospital management which has ever been published. Each of the nineteen chapters is written by a person of long experience in the management of hospitals, and who therefore can speak with authority on the subject treated.

In studying this work one finds every phase of hospital work handled in that broad-minded spirit which must recommend it to all those interested in this line of work, in connection with either large or small institutions.

The volume abounds with plates and drawings, illustrating the text and presenting valuable information regarding methods tending to economy and improved service. The appendix is a very valuable addition to the work, giving as it does a vast number of useful data concerning training school regulations, hospital dietaries and much miscellaneous information.

The book deserves and will repay careful study by all those interested in hospital management. H. C. R.

